# Inhibition of CDK9 as a therapeutic strategy for inflammatory arthritis

**DOI:** 10.1038/srep31441

**Published:** 2016-08-11

**Authors:** Annelie Hellvard, Lutz Zeitlmann, Ulrich Heiser, Astrid Kehlen, André Niestroj, Hans-Ulrich Demuth, Joanna Koziel, Nicolas Delaleu, Jan Potempa, Piotr Mydel

**Affiliations:** 1 Broegelmann Research Laboratory, Department of Clinical Science, University of Bergen, N-5021 Bergen, Norway; 2 Małopolska Centre of Biotechnology, Jagiellonian University, 30-387 Krakow, Poland; 3 Ingenium Pharmaceuticals GmbH, Fraunhoferstr. 13, 82152 Martinsried, Germany; 4 Probiodrug AG, Weinbergweg 22, Biocenter, 06120 Halle/Saale, Germany; 5 Department of Microbiology, Faculty of Biochemistry, Biophysics and Biotechnology, Jagiellonian University, 30-387 Krakow, Poland; 6 University of Louisville School of Dentistry, Department of Oral Immunology and Infectious Diseases, Louisville, KY, USA; 7 Department of Rheumatology and Inflammation Research, Sahlgrenska Academy, University of Gothenburg, Gothenburg, Sweden

## Abstract

Rheumatoid arthritis is characterised by synovial inflammation and proliferation of fibroblast-like synoviocytes. The induction of apoptosis has long been proposed as a target for proliferative autoimmune diseases, and has further been shown to act as a successful treatment of experimental models of arthritis, such as collagen-induced arthritis. Here we examined the effects of specific oral small-molecule inhibitors of the transcription regulating cyclin-dependent kinase 9 on the development and progression of collagen-induced arthritis. DBA/1 mice were immunised with bovine collagen type II and treated orally with specific CDK9 inhibitors. The effects of CDK9 inhibition on RNA levels and protein expression, apoptosis induction, caspase activation and lymphocyte phenotype were further analysed. Mice showed a significant delay in disease onset and a reduction in disease severity following treatment with CDK9 inhibitors. Inhibiting CDK9 activity in peripheral blood mononuclear cells resulted in the loss of Mcl-1 expression at both the protein and RNA levels, along with a subsequent increase in apoptosis. CDK9 specific inhibitors may be a potential alternative treatment not only of cancer, but also for autoimmune- and inflammatory diseases. Taken together, these results show that transient inhibition of CDK9 induces apoptosis in leukocyte subsets and modulates the immune response.

The hallmark of RA is inflammation of the joints due to autoimmune reactions, which over time cause irreversible damage to both cartilage and bone. Despite the high influx of inflammatory cells into RA joints and synovial hyperplasia, only low levels of apoptosis are observed^1^,^2^. This apparent dysregulation of apoptosis may enable autoreactive cells to survive and/or fail to control the number of activated effector cells, thereby promoting the development of autoimmune conditions^3^. Synovial fluid, synovial fibroblasts, and macrophages from RA patients express high levels of anti-apoptotic Bcl-2 family proteins^4^,^5^, and synovial fluid from RA patients protects neutrophils from apoptosis *in vitro* due (at least in part) to the presence of accumulated pro-inflammatory mediators and anti-apoptotic stimuli within the fluid^1^.

Recently, small-molecule inhibitors of cyclin-dependent kinases (CDKs) has been tested for their ability to induce apoptosis. CDKs are enzymes that, together with their cyclin subunits, regulate cell cycle progression (CDK1, 2, 4, and 6) and transcription (CDK7 and 9). Small-molecule compounds such as flavopiridol and roscovitine inhibit a number of different CDKs (CDK1, 2, 4, 6, 7, and 9 and CDK2, 5, 7, and 9, respectively)^6^,^7^, and various inhibitors are undergoing phase II clinical trials for the treatment of cancer. Initially, CDK inhibitors were thought to regulate proliferative diseases by inhibiting cell cycle-regulating CDKs, thereby inducing cytostasis. However, recent studies show that the most potent treatments (i.e., those that target CDK9) induce high levels of apoptosis in cancer cell lines^8^,^9^. CDK inhibitors have been used to treat inflammatory diseases in an attempt to address the over-proliferation of immune cells and fibroblasts. Treatment with the non-specific CDK inhibitor, roscovitine, induces neutrophil apoptosis by down-regulating Mcl-1 and activating caspases^10^. The pro-apoptotic effect of non-specific CDK inhibitors is mediated through inhibition of CDK9, which increases apoptosis by reducing the expression of pro-inflammatory proteins such as Mcl-1 and XIAP^8^,^11^,^12^.

Inhibition of CDK9 has a significant impact on proteins with short half-lives, e.g., anti-apoptotic proteins such as Mcl-1, which has a half-life of only a few hours^11^,^13^. Both roscovitine^10^ and flavopiridol^14^ are effective treatments for murine arthritis. However, because neither of these compounds discriminates between CDKs involved in the cell cycle and those involved in transcriptional regulation, these studies did not examine the ability of CDK9 to inhibition transcription or its subsequent effect on apoptosis.

Targeting CDK9 is a novel method of controlling immune responses without affecting the cell cycle. Garcia-Cuellar *et al.* recently showed that the CDK9 inhibitors PC585 and PC579 are efficient suppressors of mixed-lineage leukemia proliferation and that CDK9 inhibition increase the survival in a murine mixed-lineage leukemia model^15^. However, no study has yet examined whether specific CDK9 inhibitors have an effect on RA. Therefore, the aim of the present study was to examine the effects of two highly specific CDK9 inhibitors in a murine model of collagen-induced arthritis (CIA).

## Results

### Characterisation of a potent, selective inhibitor of CDK9

The two compounds (PC585 and PC579) used in the present study are specific inhibitors of CDK9^15^. Tests showed that neither compound had a significant inhibitory effect on any of 235 kinases examined when used at a concentration of 1 μM (data not shown).

### Administration of CDK9 inhibitors in murine arthritis models

**Figure 1:**
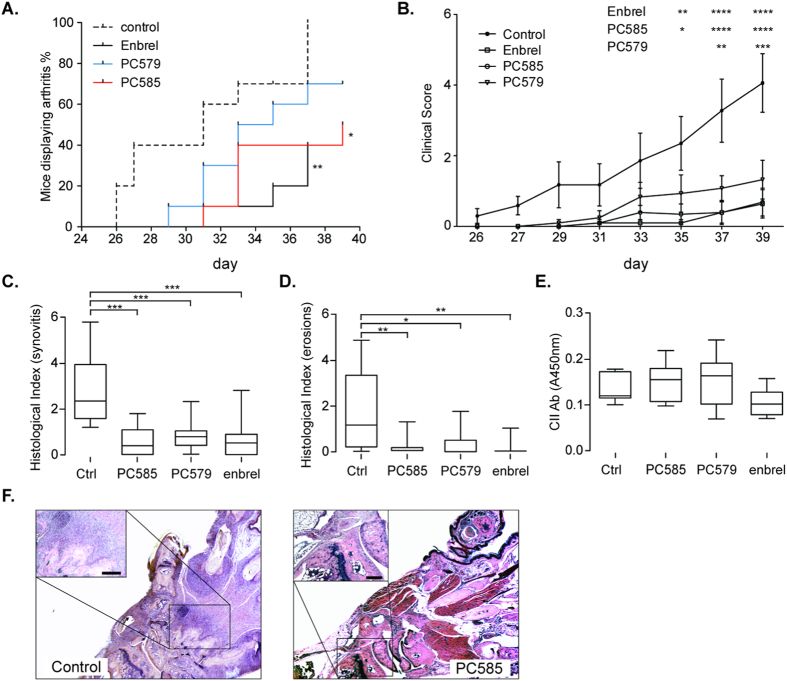
Treatment with CDK9 inhibitors ameliorates collagen induced arthritis. (**A**) Kaplan-Meier plots showing onset of arthritis during treatment with 10 mg/kg CDK9 inhibitors (PC585, n = 10 and PC579 n = 10), 100 ug anti-TNF treatment (Enbrel, n = 10) or untreated controls (n = 10). (**B**) Development of clinical signs of arthritis during the course of experiment. (**C**) Histological evaluation of synovitis and (**D**) erosions in joints (Controls, n = 10; PC585, n = 10; PC579, n = 9; Enbrel, n = 9). (**E**) Serum levels of IgG fraction of antibodies against CII at end of experiment (Controls, n = 10; PC585, n = 10; PC579, n = 9; Enbrel, n = 9). (**F**) Representative histological changes in the front paw joints of animals treated with PC585 and non-treated controls. Horizontal line and error bars represent the mean and SEM, respectively. Box and whiskers graphs show median and min to max values. Differences between groups were analyzed using log-rank test, two-way ANOVA or one-way ANOVA with Bonferronion post-test. *p < 0.05; **p < 0.01; ***p < 0.001; ****p < 0.0001.

Daily treatment with CDK9 inhibitors (PC585 and PC579; each at 10 mg/kg) had a marked impact on CIA development, progression, and severity in DBA/1 mice. We compared the effects of the two orally dosed CDK9 inhibitors with those of Enbrel (a recombinant human TNF receptor p75 Fc fusion protein commonly used to treat RA). Treatment with the CDK9 inhibitors resulted in a significant delay in disease onset. The first clinical signs of arthritis presented in control animals on Day 26, whereas animals treated with PC585 showed the first symptoms on Day 31 (p = 0.02). The effect of PC585 was comparable with that of Enbrel (p = 0.008; Fig. 1A). Treatment with PC579 did not cause a significant delay in disease onset; however, we observed a 30% reduction in disease incidence (compared with the control) at the end of the experiment (Fig. 1A). The clinical score for the control group increased rapidly up until Day 39, reaching a mean arthritis index of 4.15. By contrast, mice treated with PC585 or PC579 showed a mean arthritis index of 0.7 and 1.35, respectively (Fig. 1B). Animals treated with Enbrel showed comparatively few clinical symptoms and a mean arthritis index of 0.6 (Fig. 1B). Morphological examination of joint tissues confirmed that the CDK9 inhibitors prevented the clinical signs of CIA. When compared with that in the control group, inflammatory cell infiltration and/or thickening of the synovial membrane was markedly reduced in both treatment groups (Fig. 1C,F). In addition, the level of cartilage and bone destruction was significantly reduced in all treatment groups (PC585, *p* < 0.01; PC579, *p* < 0.05; Enbrel, *p* < 0.01) (Fig. 1D,F). Interestingly, inhibitor treatment had no impact on the level of anti-collagen type II (CII) antibodies (Fig. 1E).

### CDK9 inhibition results in the loss of Mcl-1 expression by inflammatory cells

**Figure 2:**
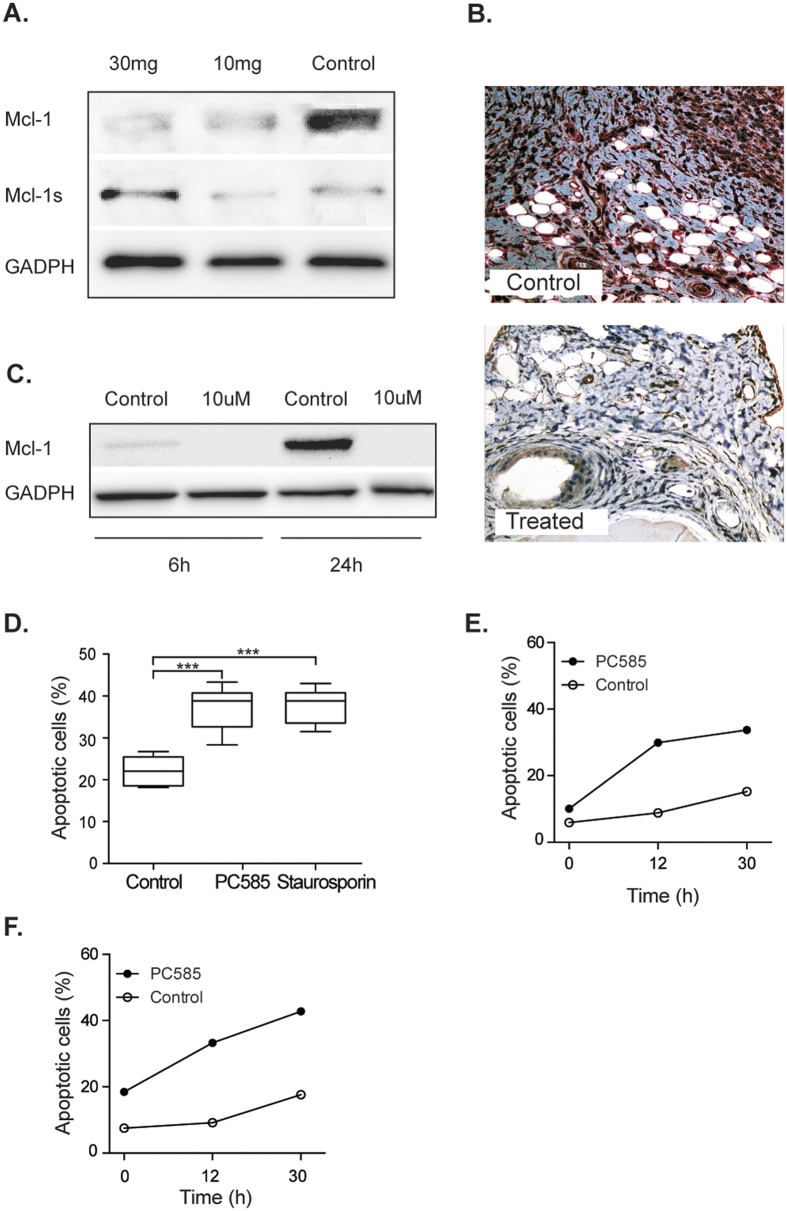
Specific CDK9 inhibitor affects apoptosis by inhibition of Mcl-1 expression. (**A**) Mcl-1 western blot on protein extract from splenocytes of arthritic mice undergoing treatment with PC585 and control. Row one and two were run on the same western blot. (**B**) Immunohistochemical staining for Mcl-1 of knee joint from CIA mice treated daily with 10 mg/kg of PC585 and control. (**C**) Western blot of protein extract of anti-CD3 activated PBMC treated with PC585 and non-treated control cells (**D**) Pro-apoptotic potential of 10 μM PC585 as compared to 2 μM staurosporin. (**E**) PBMC were pre-incubated with PC585 for 3 h and (**F**) 6 h followed by removal of PC585. One-way ANOVA with Bonferroni’s post-test was used for the statistical evaluation. ****p < 0.0001.

Non-specific CDK inhibitors impact cell survival by inducing apoptosis and by down-regulating expression of the anti-apoptotic protein, Mcl-1^10^; however, there is no direct evidence to suggest that the mechanism(s) underlying these phenomena is mediated solely by CDK9. Western blot analysis of protein extracts from the spleens of arthritic animals treated only twice weekly with PC585 (30 mg/kg and 10 mg/kg, respectively) revealed that Mcl-1 expression was down-regulated in a dose-dependent manner. Notably, there was an increase in expression of the pro-apoptotic isoform, Mcl-1s (Fig. 2A). Immunohistochemical analysis of joints from CIA mice showed that Mcl-1 expression was very low in treated animals compared with that in untreated controls. The latter showed signs of active inflammation and high expression of Mcl-1 (Fig. 2B). We further analysed Mcl-1 expression in PBMC activated with anti-CD3 *in vitro*. Treatment with CDK9 inhibitors led to reduced Mcl-1 expression as manifested by the lack of Mcl-1 protein after 6 h and 24 h (Fig. 2C). This was confirmed by reverse transcriptase PCR analysis, which showed that inhibition of CDK9 abrogated transcription of Mcl-1 (data not shown).

Taken together, these results show that specific inhibition of CDK9 in several cell types prevents the transcription of Mcl-1 and reduces its expression in peripheral lymphoid organs and disease target tissue.

### Inhibiting CDK9 induces apoptosis via a “hit-and-run” mechanism

As described above, treatment with CDK9 inhibitors led to a reduced expression of the anti-apoptotic protein Mcl-1. Therefore, we next examined the apoptotic effects of CDK9 inhibitors by staining PBMCs with Annexin V and 7AAD. Incubation with 10 μM PC585 induced apoptosis in PBMC to the same extent as staurosporine (2 μM) at 12 h (Fig. 2D). The mean percentage of Annexin V^+^ cells was 20.13% in non-treated cells and 38.28% in CDK9 inhibitor-treated cells (Fig. 2D; p < 0.0001). It is worth noting that the CDK9 inhibitors used in this study have a relatively short half-life (2 h) in plasma; therefore, to investigate whether short-term exposure is sufficient to induce the irreversible changes that lead to apoptosis, we exposed cells to 10 μM PC585 for 3 h or 6 h. The inhibitor was then removed and the number of Annexin V^+^ cells analysed at various time points thereafter. Compared with that in untreated controls, Annexin V expression by inhibitor-treated cells increased slightly at 3 h (Fig. 2E). The percent of apoptotic cells in the control samples only increased marginally up to 30 h when it reached 15.22%. By contrast, the rate of apoptosis in PBMCs treated for 3 h with PC585 increased; more than 30% of cells were Annexin V^+^ after 30 h (Fig. 2E). When cells were pre-incubated with PC585 for 6 h, 80.8% remained viable and non-apoptotic up until the point at which the inhibitor was removed. The number of apoptotic cells increased nearly 2-fold during the first 12 h following inhibitor removal, and continued to increase thereafter (42.8% of cells were Annexin V^+^ after 30 h). By contrast, only 17.65% of control cells were Annexin V^+^ (Fig. 2F).

### CDK9 inhibition increases the regulatory T cell population

**Figure 3:**
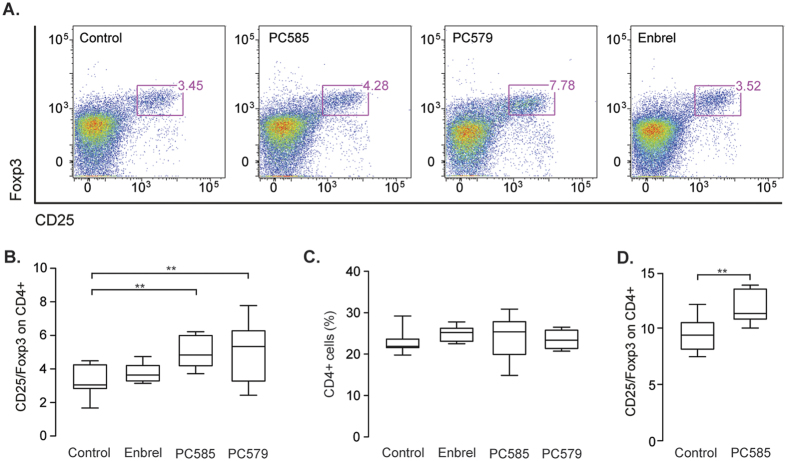
CDK9 inhibition increase percentage of splenic regulatory T cells. (**A**) Representative dot-plots of CD25 and Foxp3 expressing CD4^+^ T cells. (**B**) Flow cytometric analysis of Foxp3 and CD25 on T cells in splenocytes from arthritic mice treated daily with PC585 (n = 8), PC579 (n = 8) or Enbrel (n = 9) (Control, n = 10). (C) Percentage of CD4^+^cells in spleens of mice treated daily with PC585 (n = 8), PC579 (n = 7) or Enbrel (n = 9) (Control, n = 10) (D) CD4^+^ T cells expressing CD25 and Foxp3 in spleen of 10 healthy treated mice and 10 controls. Box and whiskers graphs show median and min to max values. Differences between two groups were analyzed using Mann-Whitney U test and One-way ANOVA with Bonferroni’s post-test was used for comparison between multiple groups. **p < 0.01.

To further examine the impact of inhibitor treatment on the immune system, we harvested splenocytes at the end of the prophylactic experiment. Flow cytometric analysis showed that the percentage of regulatory T cells (Tregs; CD4^+^CD25^+^Foxp3^+^) in spleens from arthritic mice receiving daily inhibitor treatment was significantly higher than that in control mice, even though the overall CD4^+^ population remained unchanged (Fig. 3A–C). This increase in the Treg cell population was not observed in mice receiving anti-TNF treatment. Therefore, we next treated healthy animals with 10 mg/kg of PC585 for 7 days to examine whether the CDK9 inhibitors triggered the increase in Treg cells. The results showed that the Treg cell population in the spleens of treated animals was much higher than that in non-treated healthy controls (3D).

### CDK 9 treatment prevents down-regulation of Del-1 expression in endothelial cells

**Figure 4:**
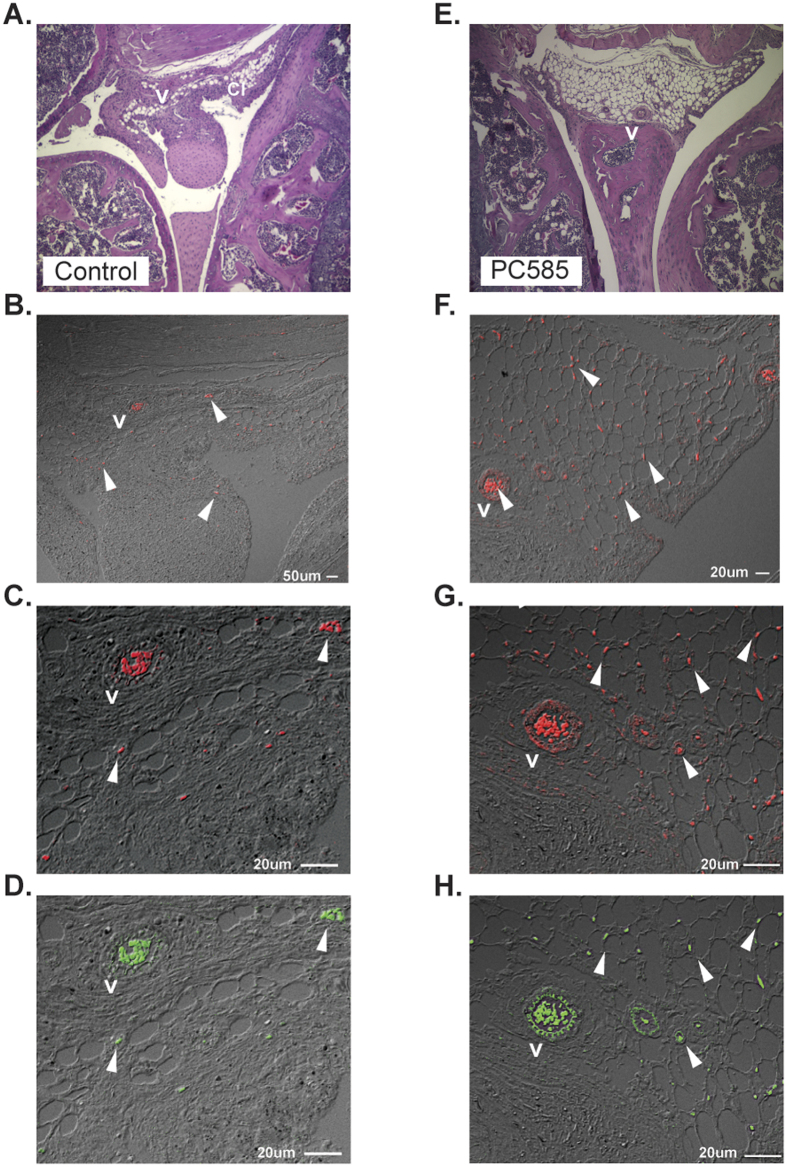
CDK 9 inhibitors protect against inflammatory Del-1 down-regulation. Representative fluorescent confocal images of slides stained for Del-1 (**B**,**C**,**F**,**G**), Ly6G (**D**,**H**) and their sequential H&E sections (**A**,**E**). Images show knee joints from arthritic DBA/1 mice treated with CDK9 inhibitor PC585 at 10 mg/kg (**E**–**H**) and arthritic controls (**A**–**D**) at day 47 of CIA. Expression is limited to endothelial cells but can be also seen also in perivascular area (V) and PMNs (arrows).

Leukocyte migration/extravasation and angiogenesis are important for the pathogenesis of RA; thus, they are interesting targets for drug development. Previous studies suggest that an endogenous leukocyte-endothelial inhibitor (called developmental endothelial locus-1; Del-1) plays a role in adhesion and leukocyte migration^16^ and apoptosis^17^, thereby contributing to the development of inflammatory diseases. Thus, we next examined the expression of Del-1 in the joints of CDK9 inhibitor-treated CIA mice and corresponding controls. Del-1 expression in synovial tissue was restricted to endothelial cells, although the protein was also detected in extravascular areas (probably due to diffusion from epithelial cells) (Fig. 4). We also found that Del-1 co-localized with neutrophils present in the joint tissue. There was a significant reduction in the expression of Del-1 protein in inflamed tissues, confirming the finding of Choi *et al.*^16^ (Fig. 4B,C). Interestingly, the levels of Del-1 were higher in joint tissues (endothelium and the perivascular areas) from mice treated with the CDK9 inhibitors (PC585 and PC579) than in non-treated animals (Fig. 4E,F). This suggests that treatment with CDK9 inhibitors leads to maintained Del-1 expression in endothelial cells, which might contribute to protection against RA by limiting LFA-1-mediated neutrophil trafficking to inflamed tissues.

### Short-term exposure to CDK9 inhibitor leads to transient transcriptional effects *in vivo*

ID family proteins regulate cell proliferation, differentiation, and angiogenesis, and their expression is up-regulated in RA patients^18^. To determine the role of CDK9 in angiogenesis, we harvested blood and spleens from CIA mice at endpoint 1, 3 and 5 h after the final administration of PC585.

**Figure 5:**
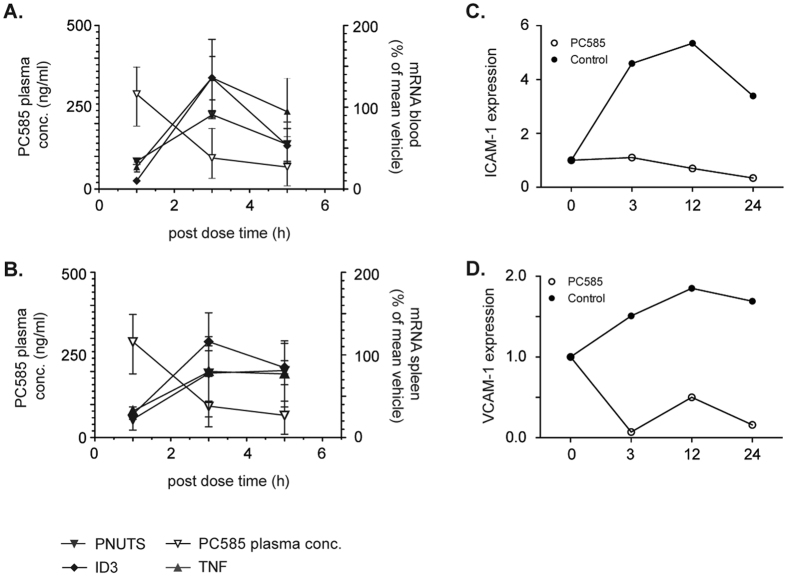
Impact of CDK9 inhibition on mRNA expression. Levels of PNUTS, ID3 and TNF mRNA were measured in (**A**) blood and (**B**) spleens of arthritic DBA/1 mice at end of experiment and superimposed on the levels of inhibitor at the increasing time points following 10 mg/kg PC585 intake (1 h, n = 4; 3 h, n =  3; 5 h, n = 3). Levels of mRNA of ICAM-1 (**C**) and VCAM-1 (**D**) in PBMC pre-incubated with PC585 for 3 h. Following incubation compound was washed out (0 h timepoint) and cells were further incubated for up to 24 h.

ID3, PNUTS and TNFα were used as biomarkers to monitor the *in vivo* transcriptional effects of CDK9 inhibition. When analysed one hour after dosing, high exposure levels of PC585 correlated with inhibition of biomarker expression to 20–30% of normal levels. All three mRNAs returned to normal levels when analysed 3 or 5 h after dosing, correlating with reduced exposure of PC585. (Fig. 5A,B). Taken together, these data show that even a transient reduction in the levels of pro-inflammatory molecules has a profound downstream impact on the development and progression of arthritis. Interestingly, when we examined the expression of VCAM-1 and ICAM-1 in PBMCs after short-term treatment with PC585 (a 3 h pre-incubation), we observed a striking down-regulation of integrin expression, which persisted after inhibitor wash-out (Fig. 5C,D).

## Discussion

To date, treatments involving small-molecule inhibitors that target CDK are aimed to control the proliferation and expansion of cancer cells^8^,^9^. As more studies use CDK inhibitors to treat inflammatory diseases, the role of transcription-regulating CDKs in the resolution of inflammation has been increasingly acknowledged^10^,^19^. Although treating CIA with flavopiridol, which inhibits CDK1, 2, 4, 6, 7, and 9, has proven to be a successful approach^14^, we hypothesised that CDK9 is the key target of flavopiridol in the treatment of RA.

When we treated CIA mice with specific CDK9 inhibitors, we observed a marked improvement in the clinical signs of arthritis. Indeed, the extent of erythema and swelling was reduced to levels observed in mice treated with a TNF inhibitor. Immunohistochemical analysis of joints from control mice revealed high levels of Mcl-1 expression; however, levels were significantly lower in the inhibitor-treated group. This is of particular interest because although Mcl-1 expression is normally tightly regulated, it is up-regulated in the joints of RA patients^4^,^5^. Compounds such as flavopiridol and roscovitine (and its more potent and selective analog, S-CR8) reduce the expression of Mcl-1 transcripts mainly by inhibiting CDK9-mediated phosphorylation of RNA polymerase II^20^. This prompted us to examine the levels of Mcl-1 in mice treated systemically with CDK9 inhibitors. Treated animals showed lower levels of splenic Mcl-1 expression than control animals, resulting in a shift towards a more pro-apoptotic environment. Studies show that the phosphatidylinositol 3-kinase (PI3-kinase)/Akt-1 and signal transducer and activator of transcription 3 (STAT3) pathways are essential for maintaining constitutive expression of Mcl-1 in macrophages^4^,^21^. CDK9 binds STAT3 upon IL-6 stimulation^22^; therefore, inhibiting CDK9 will impede the activation of this important inflammatory transcription factor, leading not only to a dampening of the inflammatory response but also a reduction in Mcl-1 expression.

When we examined the kinetics of apoptosis induction, we found that once cells had been exposed to a CDK9 inhibitor, apoptosis continued after the inhibitor was removed. The percentage of apoptotic PBMCs increased 1.71 and 2.45-fold relative to that in control cells after exposure to a CDK9 inhibitor for 3 h or 6 h, respectively. This effect lasted up to 30 h after the inhibitor was removed (the limit of the time-course measured), suggesting that a “hit-and-run” mechanism was in operation.

It is thought that RA is caused by a breakdown of self-tolerance, which in effect leads to an immune response against self-antigens. Under normal physiological conditions, Tregs cells maintain homeostasis by suppressing these autoimmune responses. Tregs protect against RA and other autoimmune diseases, although the underlying mechanism remains relatively unclear. Studies report conflicting results with respect to the number of Tregs circulating in RA patients relative to that in controls^23^,^24^,^25^. Several studies have explored the possibility of using Tregs as a potential therapeutic target in RA. There has been studies showing that induction of collagen specific oral tolerance an increase in Tregs could be observed^26^,^27^. Induction of Tregs through usage of peptides^28^, antibodies^29^, and GM-CSF^30^, has been examined in murine models of autoimmunity resulting in a significant reduction in disease severity. Interestingly, we found that the percentage of Tregs (CD4^+^CD25^+^Foxp3^+^) in animals treated with CDK9 inhibitors was higher than that in controls. This increase was not associated with changes in disease severity, as healthy mice treated with PC585 displayed a similar increase in the Tregs population. Wang *et al.* has showed that CDK inhibitors induce apoptosis of neutrophils in the presence of GM-CSF, indicating that the survival signal received during inflammation is not strong enough to counteract the pro-apoptotic effect of CDK inhibition^11^. Further, in addition to promoting survival in granulocytes GM-CSF has a clear immunomodulatory effect through its induction of Treg development both directly via the GM-CSF receptor or via tolerogenic DCs and TGF-β^30^,^31^. It has been proven that treatment with GM-CSF does expand the Treg population *in vivo*^32^ and that the presence of anti-GM-CSF antibodies may have a negative effect on inflammatory diseases such as Chron’s Disease^33^ Even though we could not identify an increase of GM-CSF in sera of inhibitor treated mice (data not shown), it may be possible that an increase of GM-CSF in local inflammatory foci or a downstream indirect effect could have caused an expansion of the Treg population.

A strong B cell response is activated in CIA mice, which results in the production of IgG antibodies directed against CII-specific epitopes^34^. These antibodies have pathogenic potential; however, their levels do not directly correlate with disease severity, as high levels can be detected in animals that show no clinical signs of disease^35^. Here, we found that treatment with CDK9 inhibitors or Enbrel had no impact on antibody levels, indicating that B cell function remained intact. This finding is interesting when we consider that CDK9 levels change significantly during B cell differentiation and activation, and that the expression of CDK9 in memory cells and activated B cells is higher than that in naïve cells^36^.

The anti-inflammatory potential of CDK9 inhibitors is supported by the evidence that CDK9 specifically regulates the pro-inflammatory transcription factor, NF-κB. NF-κB (p65 subunit) must bind to P-TEFb to induce transcription-elongation^37^. Interestingly, gene silencing and treatment with the non-specific CDK inhibitor, flavopiridol, inhibited the recruitment of NF-κB in human endothelial cells, resulting in a significant reduction in ICAM-1 expression, which is crucial for lymphocyte recruitment to inflamed tissues^38^. Our data confirm these results, *in vitro* experiments revealed that even a short exposure to a CDK9 inhibitor led to a significant reduction in the expression of mRNA for ICAM-1 and VCAM-1. Leukocyte migration/extravasation and angiogenesis are very important for the pathogenesis of RA^39^,^40^,^41^; therefore, they are interesting targets for drug development. Previous studies suggest that Del-1, which is implicated in angiogenesis, apoptosis, adhesion, migration, and proliferation^38^,^42^ might be regulated by NF-κB^43^. When expressed on the endothelium, Del-1 competes with ICAM-1 for binding to LFA-1, thereby inhibiting leukocyte adhesion and subsequent migration through the vessel wall. Animal studies show that by inhibiting leukocyte recruitment, Del-1 suppresses both the duration and magnitude of the inflammatory response^16^. Here, we demonstrated that Del-1 expression in the endothelium is dependent upon CDK9 and it is tempting to speculate that the regulatory mechanism acts via inactivation of NF-κB. Examination of inflamed joint tissues revealed significant down-regulation of Del-1 expression, which was reversed by treatment with a CDK9 inhibitor. It has been shown that Del-1 reduces IL-17-mediated bone loss in a periodontitis model by inhibiting IL-17-dependent neutrophil recruitment to inflamed tissues^44^. Both Del-1 and IL-17 are reciprocally cross regulated; indeed, Del-1-mediated down-regulation of IL-17 is one of the most important factors that causes cartilage and bone destruction within the joint. Therefore, IL-17 inhibitors are an attractive putative RA treatment. The finding that CDK9 inhibitors up-regulate Del-1 expression in joint endothelial cells may suggest yet another mechanism that protects against joint inflammation that require further investigation.

## Material and Methods

### Animals

Male DBA/1 (Taconic Europe A/S and Harlan Laboratories) aged 6–8 weeks old were kept under standard environmental conditions and had free access to laboratory chow and water. Male DBA/1 mice aged 13 weeks were used as healthy controls for confocal staining. Experiments were approved by the Animal Research Ethical Committee of Gothenburg University, and The UK Home Office and carried out in accordance with the approved guidelines.

### Induction of Arthritis

Bovine Collagen type II (Chondrex) were emulsified with equal volume of Freund’s complete adjuvant and mice were immunized with 100 μg on day 0 and boosted with 100 μg CII in Freund’s incomplete adjuvant on day 21. When analysing the RNA expression of Pnuts, ID3, TNF and PC585 in blood and spleens mice were immunized with 200 μg Bovine Collagen type II (MD Biosciences) in Freund’s complete adjuvant and boosted on day 21 with 200 μg collagen in PBS *i.p,* followed by 10 μg LPS on day 22.

### Treatment with CDK9 inhibitors

DBA/1 mice were treated with specific CDK9 inhibitors, PC585 and PC579 at 10 mg/kg. The inhibitors were sonicated in 0.8% methyl cellulose (Methocel E4M, Dow) and provided to mice through oral gavage. *i.p* injections of 4 mg/kg Enbrel (etanercept, Wyeth Europe) dosed biweekly was used as a reference. Treatment started three days prior induction of arthritis. For the mRNA expression study daily treatment with 10 mg/kg of PC585 was initiated at onset of arthritis and sustained for 21 days, until end of experiment. Healthy NMRI mice were treated for 7 consecutive days with PC585 to assess the effect of treatment on T cell populations.

### Clinical evaluation of CIA

Clinical scoring of collagen induced arthritis was performed by an examiner blinded and assessed through scoring from 0–3. Swelling and erythema of only a toe or finger was scored as 0.5. In the event of culling of an individual mouse the last observed score was carried forward.

### Histological evaluation

Hematoxylin and Eosin stained sections were evaluated for synovitis and erosion of bone and/or cartilage by a blinded examiner. Joints in knees, ankles, toes, elbows, wrists and fingers were scored on a scale from 0–3 (1, mild; 2, moderate; and 3, severe) where half points were allowed. The histological score was calculated by dividing the total score per mouse by paws. Occasionally histological sections were not possible to score and they were therefore excluded.

### Flow cytometric phenotype analysis

Spleens from DBA/1 and NMRI mice were excised and pushed through a 70 μm mesh to obtain a single cell solution. Splenocytes from DBA/1 mice were frozen in 10% DMSO/FCS until FACS analysis. Cells were pelleted, incubated with Fc-block (anti-CD16/CD32, BD Pharmingen) and stained with following antibodies conjugated to either, PE, APC, APC-H7: CD25 (PC61.5, eBioscience) CD19 (1D3, eBioscience) and CD4 (GK1.5, BD Pharmingen). Following surface stain cells were fixed and permeabilized for staining of Foxp3 (FJK-16a, eBioscience) or isotype control (eBR2a, eBioscience). Cells were collected using a FACSCantoII equipped with FACSDiva software. Analysis was performed using FlowJo software and fluorochrome minus one (FMO) controls were used for gating when necessary.

### Flow cytometric analysis of apoptosis

Human peripheral mononuclear cells (PBMC) were obtained by gradient centrifugation on lymphoprep (Axis-shield) and incubated in 37 °C for 12 h with addition of 10 μM PC585 in complete RPMI1640 media. two μM staurosporine was used as positive control. For further kinetic evaluation PBMC was incubated with 10 μM PC585 for 3 or 6 h in complete RPMI media. Following incubation media was replaced and cells were incubated further. Cells were harvested at point of washout (0 h) and additional time-points spanning between 12–30 h. The cells were washed with Annexin V binding buffer and stained with Annexin V-eFlour 450 and 7AAD (eBioscience) and analysed through flowcytometry. Flow cytometry dot plots are shown in Supplementary figure 1.

### Western blot

PBMC were seeded on anti-CD3 (RnD Systems) coated 12-well plate in complete RPMI for 6 or 24 h in the presence of 10 μM PC585. PBMC, as well as splenocytes from arthritic mice treated biweekly with PC585 and controls, were lysed with RIPA buffer. Samples were resolved on 4–15% Tris-HCl gel (Bio-Rad) and transferred to PVDF membrane (Bio-Rad). Following blocking with skim milk membranes were incubated with rabbit anti-human/anti-mouse Mcl-1 antibody (Y37, Abcam) as primary and swine anti-rabbit HRP conjugated antibody (Dako) as secondary antibody. Mouse anti-rabbit GAPDH (6C5, HyTest) were used as a loading control. Blots were developed using ECL detection (Western Blotting Detection Reagents; Amersham Biosciences) and images were acquired using Bio-Rad ChemiDoc XRS + system. Adobe Photoshop CS6 was used for post image processing.

### Quantitative PCR

RNA (0.1 μg) was reversely transcribed into cDNA using random primers (Roche) and Superscript III (Life Technologies). Quantitative PCR was performed in a Rotorgene3000 (Corbett Research) using the Rotor-Gene SYBR Green PCR kit (Qiagen) and specific primers for Pnuts, Id3, Mcl1, Icam1, and Vcam all synthesized by Metabion (Table 1). Relative amounts of gene expression were determined with the Rotorgene software version 6.1 in comparative quantitation mode. Normalization was done against the most stably expressed reference gene Ywhaz (NM_011740.2) identified using Normfinder^45^. The PCR was verified by product melting curves and single amplicons were confirmed by agarose gel electrophoresis.

### Immunohistochemistry

Joints sections were cut and subjected to antigen retrieval in decloaker chamber in Diva Decloaker (Histolab) following deparaffination. Sections were blocked with Protein Block (Dako) for 30 min before incubation with Rabbit anti-human/anti-mouse Mcl-1 antibody (Y37, abcam). Peroxidase activity was blocked with 3% H_2_O_2_. Slides were incubated with biotinylated swine anti-rabbit IgG (Dako) and developed using ABC detection system with AEC substrate (Vectastain). Sections were counterstained with hematoxylin and mounted.

### Detection of serum antibodies

Antibodies against CII were detected by coating 96-well plates with bovine CII (Chondrex) at the concentration 1 ug/ml. Samples were serial diluted and incubated for 2 h in RT following blocking with 0.5% BSA. Secondary antibodies were purchased from Jackson Immunoresearch. Plates were developed with TMB substrate reagent set (BD), stopped with 1 M H_2_SO_4_ and read at 450 nm.

### Confocal microscopy

Sections from mice treated with inhibitor twice weekly were stained with antibodies against mouse Ly6G (RB6-8C5, FITC- conjugate, LifeSpan BioSciences) or with antibodies against human/mouse Del-1 (ProteinTech). Where necessary, staining involved the use of a secondary reagent (AlexaFluor594-conjugated goat anti-rabbit IgG, Molecular Probes). Images were captured using a laser-scanning confocal microscope (Olympus FV1000). Of note, because Del-1 expression decreases with age^46^, all immunochemical staining was performed on animals of similar age.

### Statistical analysis

Statistical differences were performed using two-way ANOVA or one-way ANOVA with Bonferroni post-test for multiple comparisons. Kaplan-Meyer graphs were analysed using log-rank test with Bonferroni post-test. Mann-Whitney U-test was used for statistical analysis between two groups. All statistical analyses were performed using GraphPad Prism, version 5.0d or 6.0b for Mac, a P value of <0.05 was considered statistically significant.

## Additional Information

**How to cite this article**: Hellvard, A. *et al.* Inhibition of CDK9 as a therapeutic strategy for inflammatory arthritis. *Sci. Rep.* **6**, 31441; doi: 10.1038/srep31441 (2016).

## Supplementary Material

Supplementary Information

## Figures and Tables

**Table 1:** Primers used for quantitative PCR.

**mPNUTS-F**	GGTCCTGGTTGGTTGCTTTA	**mPNUTS-R**	TCTTTCGTGCCTCCTTCATT	NM_175934.3|
**mID3-F**	ACTCAGCTTAGCCAGGTGGA	**mID3-R**	AAGCTCCTCTTGTCCTTGGAG	BC145871
**mTNFa-F**	GAACTGGCAGAAGAGGCACT	**mTNFa-R**	AGGGTCTGGGCCATAGAACT	NM_013693.1|
**mYWHAZ-F**	TGAAGCCATTGCTGAACTTG	**mYWHAZ-R**	GTTGGAAGGCCGGTTAATTT	NM_011740.2|
**hICAM1-F**	GGCTGGAGCTGTTTGAGAAC	**hICAM-1R**	TCACACTGACTGAGGCCTTG	NM_000201.1
**hVCAM1-F**	ATGGGAAGGTGACGAATGAG	**hVCAM1-R**	ATCTCCAGCCTGTCAAATGG	NM_001078.2
**hYWHAZ-F**	AGCAGGCTGAGCGATATGAT	**hYWHAZ-R**	TCTCAGCACCTTCCGTCTTT	NM_003406.2

## References

[b1] Ottonello L. *et al.* Synovial fluid from patients with rheumatoid arthritis inhibits neutrophil apoptosis: role of adenosine and proinflammatory cytokines. Rheumatol. Oxf. Engl. 41, 1249–1260 (2002). 10.1093/rheumatology/41.11.1249 12421997

[b2] Raza K. *et al.* Synovial fluid leukocyte apoptosis is inhibited in patients with very early rheumatoid arthritis. Arthritis Res. Ther. 8, R120 (2006). 16859518 10.1186/ar2009 PMC1779404

[b3] Solary E., Dubrez L. & Eymin B. The role of apoptosis in the pathogenesis and treatment of diseases. Eur. Respir. J. 9, 1293–1305 (1996). 10.1183/09031936.96.09061293 8804951

[b4] Liu H. *et al.* Regulation of Mcl-1 expression in rheumatoid arthritis synovial macrophages. Arthritis Rheum. 54, 3174–3181 (2006). 10.1002/art.22132 17009247

[b5] Liu H. *et al.* Mcl-1 is essential for the survival of synovial fibroblasts in rheumatoid arthritis. J. Immunol. Baltim. Md 1950 175, 8337–8345 (2005). 10.4049/jimmunol.175.12.8337 16339575

[b6] Sedlacek H. H. Mechanisms of action of flavopiridol. Crit. Rev. Oncol. Hematol. 38, 139–170 (2001). 10.1016/s1040-8428(00)00124-4 11311660

[b7] Chao S. H. & Price D. H. Flavopiridol inactivates P-TEFb and blocks most RNA polymerase II transcription *in vivo*. J. Biol. Chem. 276, 31793–31799 (2001). 11431468 10.1074/jbc.M102306200

[b8] Cai D., Latham V. M., Zhang X. & Shapiro G. I. Combined depletion of cell cycle and transcriptional cyclin-dependent kinase activities induces apoptosis in cancer cells. Cancer Res. 66, 9270–9280 (2006). 16982772 10.1158/0008-5472.CAN-06-1758

[b9] MacCallum D. E. *et al.* Seliciclib (CYC202, R-Roscovitine) induces cell death in multiple myeloma cells by inhibition of RNA polymerase II-dependent transcription and down-regulation of Mcl-1. Cancer Res. 65, 5399–5407 (2005). 10.1158/0008-5472.CAN-05-0233 15958589

[b10] Rossi A. G. *et al.* Cyclin-dependent kinase inhibitors enhance the resolution of inflammation by promoting inflammatory cell apoptosis. Nat. Med. 12, 1056–1064 (2006). 10.1038/nm1468 16951685

[b11] Wang K. *et al.* Cyclin-dependent kinase 9 activity regulates neutrophil spontaneous apoptosis. PloS One 7, e30128 (2012). 22276149 10.1371/journal.pone.0030128 PMC3261871

[b12] Liu X. *et al.* CDKI-71, a novel CDK9 inhibitor, is preferentially cytotoxic to cancer cells compared to flavopiridol. Int. J. Cancer J. Int. Cancer 130, 1216–1226 (2012). 10.1002/ijc.26127 21484792

[b13] Akgul C., Moulding D. A., White M. R. & Edwards S. W. *In vivo* localisation and stability of human Mcl-1 using green fluorescent protein (GFP) fusion proteins. FEBS Lett. 478, 72–76 (2000). 10.1016/s0014-5793(00)01809-3 10922472

[b14] Sekine C. *et al.* Successful treatment of animal models of rheumatoid arthritis with small-molecule cyclin-dependent kinase inhibitors. J. Immunol. Baltim. Md 1950 180, 1954–1961 (2008). 10.4049/jimmunol.180.3.1954 18209094

[b15] Garcia-Cuellar M.-P. *et al.* Efficacy of cyclin-dependent-kinase 9 inhibitors in a murine model of mixed-lineage leukemia. Leukemia 28, 1427–1435 (2014). 10.1038/leu.2014.40 24445865

[b16] Choi E. Y. *et al.* Del-1, an endogenous leukocyte-endothelial adhesion inhibitor, limits inflammatory cell recruitment. Science 322, 1101–1104 (2008). 10.1126/science.1165218 PMC2753175 19008446

[b17] Kitano H., Kokubun S. & Hidai C. The extracellular matrix protein Del1 induces apoptosis via its epidermal growth factor motif. Biochem. Biophys. Res. Commun. 393, 757–761 (2010). 10.1016/j.bbrc.2010.02.076 20171188

[b18] Sakurai D., Yamaguchi A., Tsuchiya N., Yamamoto K. & Tokunaga K. Expression of ID family genes in the synovia from patients with rheumatoid arthritis. Biochem. Biophys. Res. Commun. 284, 436–442 (2001). 11394898 10.1006/bbrc.2001.4974

[b19] Leitch A. E. *et al.* Cyclin-dependent kinases 7 and 9 specifically regulate neutrophil transcription and their inhibition drives apoptosis to promote resolution of inflammation. Cell Death Differ. 19, 1950–1961 (2012). 10.1038/cdd.2012.80 PMC3504709 22743999

[b20] Bettayeb K. *et al.* CR8, a potent and selective, roscovitine-derived inhibitor of cyclin-dependent kinases. Oncogene 27, 5797–5807 (2008). 18574471 10.1038/onc.2008.191

[b21] Liu H., Perlman H., Pagliari L. J. & Pope R. M. Constitutively activated Akt-1 is vital for the survival of human monocyte-differentiated macrophages. Role of Mcl-1, independent of nuclear factor (NF)-kappaB, Bad, or caspase activation. J. Exp. Med. 194, 113–126 (2001). 11457886 10.1084/jem.194.2.113 PMC2193455

[b22] Hou T., Ray S. & Brasier A. R. The functional role of an interleukin 6-inducible CDK9.STAT3 complex in human gamma-fibrinogen gene expression. J. Biol. Chem. 282, 37091–37102 (2007). 10.1074/jbc.M706458200 17956865

[b23] Liu M.-F., Wang C.-R., Fung L.-L., Lin L.-H. & Tsai C.-N. The presence of cytokine-suppressive CD4+CD25+ T cells in the peripheral blood and synovial fluid of patients with rheumatoid arthritis. Scand. J. Immunol. 62, 312–317 (2005). 10.1111/j.1365-3083.2005.01656.x 16179019

[b24] Van Amelsfort J. M. R., Jacobs K. M. G., Bijlsma J. W. J., Lafeber F. P. J. G. & Taams L. S. CD4(+)CD25(+) regulatory T cells in rheumatoid arthritis: differences in the presence, phenotype, and function between peripheral blood and synovial fluid. Arthritis Rheum. 50, 2775–2785 (2004). 10.1002/art.20499 15457445

[b25] Xiao H., Wang S., Miao R. & Kan W. TRAIL is associated with impaired regulation of CD4+CD25- T cells by regulatory T cells in patients with rheumatoid arthritis. J. Clin. Immunol. 31, 1112–1119 (2011). 21732015 10.1007/s10875-011-9559-x

[b26] Yoshinari O. *et al.* Water-soluble undenatured type II collagen ameliorates collagen-induced arthritis in mice. J. Med. Food 16, 1039–1045 (2013). 10.1089/jmf.2013.2911 24175655

[b27] Faria A. M. C. & Weiner H. L. Oral tolerance: therapeutic implications for autoimmune diseases. Clin. Dev. Immunol. 13, 143–157 (2006). 10.1080/17402520600876804 PMC2270752 17162357

[b28] Chen G. *et al.* The therapeutic effect of vasoactive intestinal peptide on experimental arthritis is associated with CD4+CD25+ T regulatory cells. Scand. J. Immunol. 68, 572–578 (2008). 19055696 10.1111/j.1365-3083.2008.02178.x

[b29] Notley C. A., McCann F. E., Inglis J. J. & Williams R. O. ANTI-CD3 therapy expands the numbers of CD4+ and CD8+ Treg cells and induces sustained amelioration of collagen-induced arthritis. Arthritis Rheum. 62, 171–178 (2010). 20039431 10.1002/art.25058

[b30] Kared H. *et al.* Role of GM-CSF in tolerance induction by mobilized hematopoietic progenitors. Blood 112, 2575–2578 (2008). 18617637 10.1182/blood-2008-02-140681

[b31] Bhattacharya P. *et al.* GM-CSF: An immune modulatory cytokine that can suppress autoimmunity. Cytokine 75, 261–271 (2015). 26113402 10.1016/j.cyto.2015.05.030 PMC4553090

[b32] Rowin J. *et al.* Granulocyte Macrophage Colony Stimulating Factor Treatment of a Patient in Myasthenic Crisis: Effects on Regulatory T cells. Muscle Nerve 46, 449–453 (2012). 22907239 10.1002/mus.23488 PMC3428740

[b33] Gathungu G. *et al.* Granulocyte-Macrophage Colony-Stimulating Factor Auto-Antibodies: A Marker of Aggressive Crohn’s Disease. Inflamm. Bowel Dis. 19, 1671–1680 (2013). 23749272 10.1097/MIB.0b013e318281f506 PMC3707315

[b34] Holmdahl R. *et al.* Incidence of arthritis and autoreactivity of anti-collagen antibodies after immunization of DBA/1 mice with heterologous and autologous collagen II. Clin. Exp. Immunol. 62, 639–646 (1985). PMC1577464 4085150

[b35] Reife R. A., Loutis N., Watson W. C., Hasty K. A. & Stuart J. M. SWR mice are resistant to collagen-induced arthritis but produce potentially arthritogenic antibodies. Arthritis Rheum. 34, 776–781 (1991). 2053925 10.1002/art.1780340621

[b36] De Falco G. *et al.* Cdk9/Cyclin T1 complex: a key player during the activation/differentiation process of normal lymphoid B cells. J. Cell. Physiol. 215, 276–282 (2008). 18205180 10.1002/jcp.21311

[b37] Barboric M., Nissen R. M., Kanazawa S., Jabrane-Ferrat N. & Peterlin B. M. NF-kappaB binds P-TEFb to stimulate transcriptional elongation by RNA polymerase II. Mol. Cell 8, 327–337 (2001). 11545735 10.1016/s1097-2765(01)00314-8

[b38] Schmerwitz U. K. *et al.* Flavopiridol protects against inflammation by attenuating leukocyte-endothelial interaction via inhibition of cyclin-dependent kinase 9. Arterioscler. Thromb. Vasc. Biol. 31, 280–288 (2011). 21088252 10.1161/ATVBAHA.110.213934

[b39] Kavanaugh A. F. *et al.* Treatment of refractory rheumatoid arthritis with a monoclonal antibody to intercellular adhesion molecule 1. Arthritis Rheum. 37, 992–999 (1994). 7912930 10.1002/art.1780370703

[b40] Paleolog E. M. & Miotla J. M. Angiogenesis in arthritis: role in disease pathogenesis and as a potential therapeutic target. Angiogenesis 2, 295–307 (1998). 14517450 10.1023/a:1009229508096

[b41] Koch A. E., Halloran M. M., Haskell C. J., Shah M. R. & Polverini P. J. Angiogenesis mediated by soluble forms of E-selectin and vascular cell adhesion molecule-1. Nature 376, 517–519 (1995). 10.1038/376517a0 7543654

[b42] Rezaee M., Penta K. & Quertermous T. Del1 mediates VSMC adhesion, migration, and proliferation through interaction with integrin alpha(v)beta(3). Am. J. Physiol. Heart Circ. Physiol. 282, H1924–H1932 (2002). 11959660 10.1152/ajpheart.00921.2001

[b43] Kim H. *et al.* p53 regulates the transcription of the anti-inflammatory molecule developmental endothelial locus-1 (Del-1). Oncotarget 4, 1976–1985 (2013). 24192518 10.18632/oncotarget.1318 PMC3875763

[b44] Eskan M. A. *et al.* The leukocyte integrin antagonist Del-1 inhibits IL-17-mediated inflammatory bone loss. Nat. Immunol. 13, 465–473 (2012). 22447028 10.1038/ni.2260 PMC3330141

[b45] Andersen C. L., Jensen J. L. & Ørntoft T. F. Normalization of Real-Time Quantitative Reverse Transcription-PCR Data: A Model-Based Variance Estimation Approach to Identify Genes Suited for Normalization, Applied to Bladder and Colon Cancer Data Sets. Cancer Res. 64, 5245–5250 (2004). 15289330 10.1158/0008-5472.CAN-04-0496

[b46] Shin J. *et al.* Expression and function of the homeostatic molecule Del-1 in endothelial cells and the periodontal tissue. Clin. Dev. Immunol. 2013, 617809 (2013). 24416060 10.1155/2013/617809 PMC3876683

