# Supplementary material for: Inhibition of CDK9 as a therapeutic strategy for inflammatory arthritis
Source: Sci Rep. 2016 Aug 11;6:31441. doi: 10.1038/srep31441 (PMC4980610; doi:10.1038/srep31441)
Supplement: Supplementary Information [file srep31441-s1.pdf]

Inhibition of CDK9 as a therapeutic strategy for inflammatory arthritis

Annelie Hellvard, Lutz Zeitlmann, Ulrich Heiser, Astrid Kehlen, André Niestroj, Hans-Ulrich Demuth, Joanna Koziel, Nicolas Delaleu, Jan Potempa and Piotr Mydel

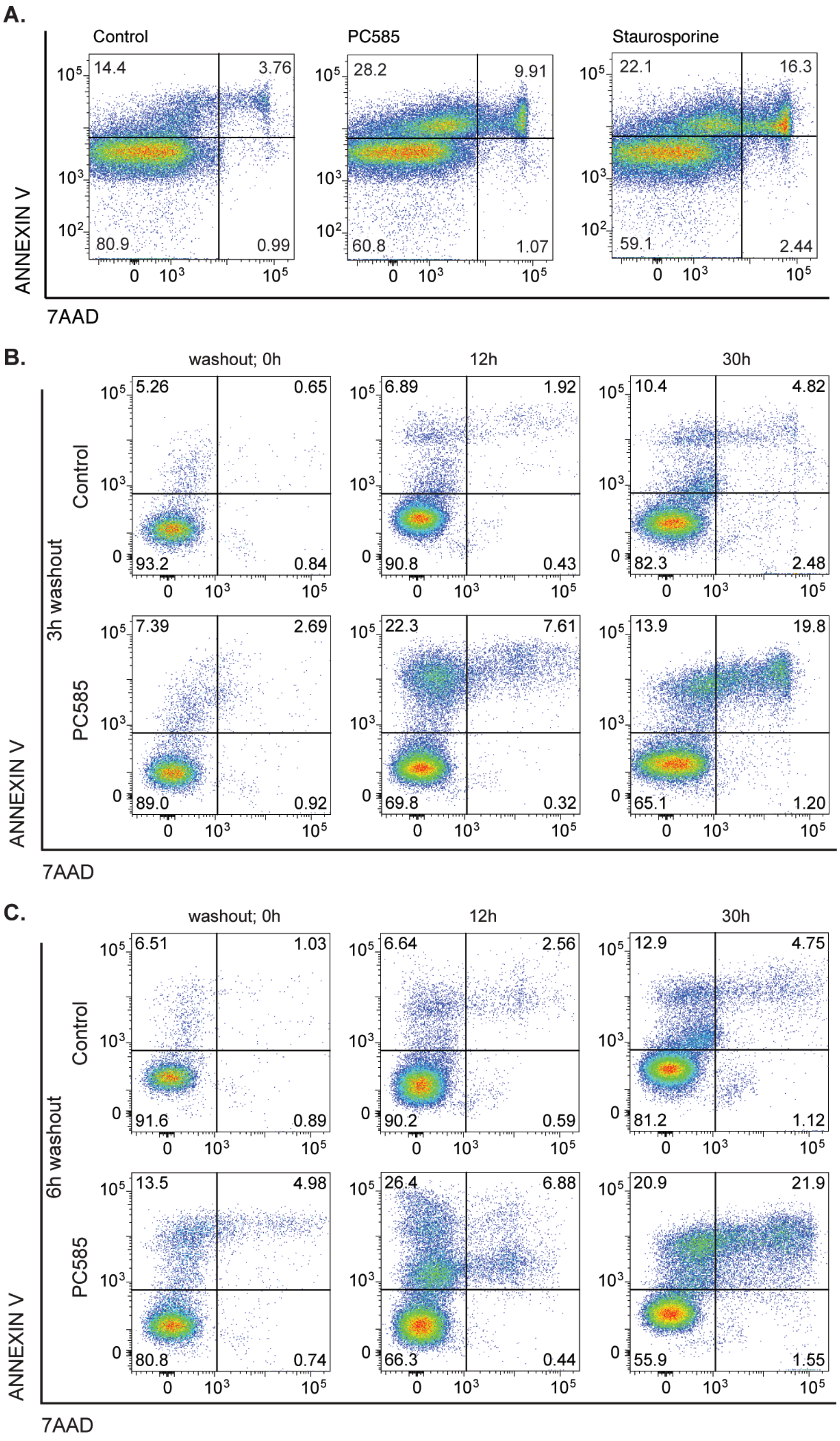

Supplementary figure 1. Flow cytometry dot plots representative for data presented in figure 3D (A). Flow cytometry dot plots of data presented in figure 2E (B) and 2F (C).
